# It’s Only a Part of the Story: Analytical Investigation of the Inks and Dyes Used in the *Privilegium Maius*

**DOI:** 10.3390/molecules24122197

**Published:** 2019-06-12

**Authors:** Elisa Calà, Fabio Gosetti, Monica Gulmini, Ilaria Serafini, Alessandro Ciccola, Roberta Curini, Annalisa Salis, Gianluca Damonte, Kathrin Kininger, Thomas Just, Maurizio Aceto

**Affiliations:** 1 Dipartimento di Scienze e Innovazione Tecnologica, Università degli Studi del Piemonte Orientale, viale T. Michel, 11-15121 Alessandria, Italy; elisa.cala@uniupo.it (E.C.); fabio.gosetti@uniupo.it (F.G.); 2 Dipartimento di Chimica, Università degli Studi di Torino, via P. Giuria, 7-10125 Torino, Italy; monica.gulmini@unito.it; 3 Dipartimento di Chimica, Università di Roma “La Sapienza”, Piazzale Aldo Moro 5, 00185 Roma, Italy; ilaria.serafini@uniroma1.it (I.S.); alessandro.ciccola@uniroma1.it (A.C.); roberta.curini@uniroma1.it (R.C.); 4 Centre of Excellence for Biomedical Research (CEBR), Università degli Studi di Genova, viale Benedetto XV, 5-16132 Genova, Italy; annalisa.salis@unige.it (A.S.); gianluca.damonte@unige.it (G.D.); 5 Österreichisches Staatsarchiv, Haus-, Hof- und Staatsarchiv, Minoritenplatz, 1-1010 Wien, Austria; kathrin.kininger@oesta.gv.at (K.K.); thomas.just@oesta.gv.at (T.J.)

**Keywords:** alizarin, Austria, dyes, forgery, fibre optic (FORS), inks, madder, manuscripts, orchil, Surface Enhanced Raman Spectroscopy (SERS)

## Abstract

The *Privilegium maius* is one of the most famous and spectacular forgeries in medieval Europe. It is a set of charters made in the 14th century upon commitment by Duke Rudolf IV, a member of the Habsburg family, to elevate the rank and the prestige of his family. These five charters, now kept at the Österreichisches Staatsarchiv in Vienna, have been subjected to a thorough interdisciplinary study in order to shed light on its controversial story. The charters are composed of pergamenaceous documents bound to wax seals with coloured textile threads. The present contribution concerns the characterisation of the inks used for writing and of the dyes used to colour to the threads: Are they compatible with the presumed age of the charters? Though showing only a part of the whole story of the charters, dyes analysis could contribute in assessing their complex history from manufacturing to nowadays. The dyes were characterised with non-invasive in situ measurements by means of fibre optic (FORS) and with micro-invasive measurements by means of Surface Enhanced Raman Spectroscopy (SERS) and High-Performance Liquid Chromatography with Mass Spectrometry (HPLC-MS) analysis. The results showed that the threads of four of the charters (three dyed with madder, one with orchil) were apparently coloured at different dyeing stages, then re-dyed in the 19–20th century.

## 1. Introduction

### The History of the Privilegium Maius

The *Privilegium maius* is one of the most famous and most spectacular forgeries in medieval Europe. It is a set of five pergamenaceous charters dating to the 14th century AD with a highly controversial story. In 1156, the Emperor of the Holy Roman Empire Frederick I called Barbarossa issued the *Privilegium minus*, a charter with a decree that elevated Austria to the rank of Duchy ruled by the Babenberg family. The Babenbergs were successively replaced by the Habsburg family in 1278 as the rulers of the Duchy of Austria. In 1356, Charles IV, Emperor of the Holy Roman Empire, issued the *Goldene Bulle*, defining the constitutional structure of the Empire and establishing the list of the seven Prince-electors, among which the Duke of Austria was not comprised. Possibly two to three years later, Duke Rudolf IV, member of the Habsburg family, who was disappointed at the exclusion of the Habsburgs from the list of the Prince-electors, commissioned a forgery of the *Privilegium minus* and of four other charters, trying to elevate the rank and the prestige of his family; this set of five charters is historically known as *Privilegium maius*. They are now kept in the Haus-, Hof- und Staatsarchiv Department of the Österreichisches Staatsarchiv in Vienna [1]. For almost 200 years it has been known that the documents are false: Due to their inner and outer characteristics, historians have been able to see through the, albeit, excellent forgeries.

To shed light on the controversial story of the *Privilegium maius* charters, these documents have been recently subjected to a diagnostic study that involved the different parts they are made of: The parchment support, the text they were written with, the beeswax seals linked to the document and the coloured threads that link the seals to the document.

**Figure 1:**
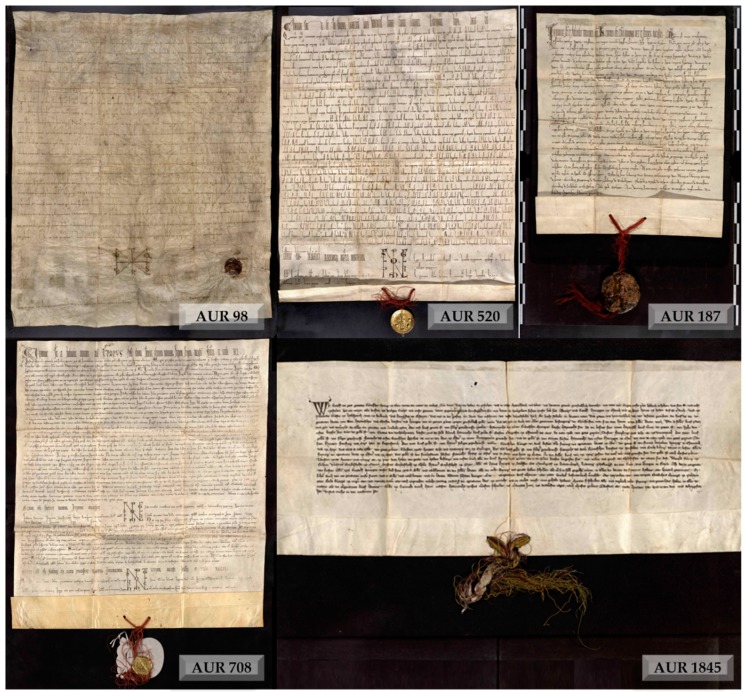
The five charters constituting the *Privilegium maius*.

Among the five charters composing the *Privilegium maius*, namely AUR 98, AUR 187, AUR 520, AUR 708, and AUR 1845, all but the first have coloured threads linking seals to the parchments (Figure 1).

The present study is focused on the dyes used to colour the threads, with the aim of disclosing whether the materials employed are actually compatible with the presumed age of the charters. The investigation also aims at shedding light on how and when the dyes were applied. In addition, the inks used for the text of the documents have been also considered. Although a study on the inks and dyes shows only a part of the charters’ complex story, these analyses could contribute in assessing the sequence of their manufacture. To complement the study and provide comparative data, other 13–14th century charters kept at Österreichisches Staatsarchiv (AUR 97, AUR 1359 VIII 10, Vidimus AUR 1360 VII 11, AUR 1792, and AUR 1844) were analysed as well.

Firstly, non-invasive analysis was performed by means of UV–visible diffuse reflectance spectrophotometry with fibre optic (FORS) and molecular spectrofluorimetry with fibre optic (FOMF) to preliminarily identify of the dyes colouring the threads and to obtain information on the kind of the ink employed in the text. These measurements were carried out in situ on the charters at the Österreichisches Staatsarchiv. Further on, samples from the threads were taken to perform Raman analysis—both in normal mode and Surface Enhanced Raman Spectroscopy (SERS)—and micro-destructive measurements by means of High-Performance Liquid Chromatography coupled with Diode Array Detection and Mass Spectrometry (HPLC-DAD-MS), in order to have a full characterisation of the dyes.

## 2. Results

### 2.1. Analysis of the Inks

The charters of the *Privilegium maius* and the complementary documents contain only black inks (the verso of AUR 1360 VII 11 contain a few lines of red writing that we identified as composed of cinnabar or vermilion, a typical pigment used in the Middle Ages for rubrics). The analysis of the black inks was carried out using FORS.

Historically, the main types of black ink were (1) *carbon-based* inks, (2) *iron gall* inks (IGI), (3) *sepia* inks and (4) *bistre* inks, with the first two types being by far the most popular. Carbon-based inks were the first to be created, having been in use since at least 2500 BCE in East Asia. IGI were introduced much later, being known since the 4th century AD; the oldest occurrences identified are in the *Vercelli Gospels* (Biblioteca Capitolare, Vercelli, ms. A) [2] and in the *Codex Sinaiticus* (British Library, London) [3]. Within a few centuries, IGI almost totally replaced the carbon-based inks all throughout the Middle Ages and until the introduction of synthetic inks produced from petrol derivatives.

**Figure 2:**
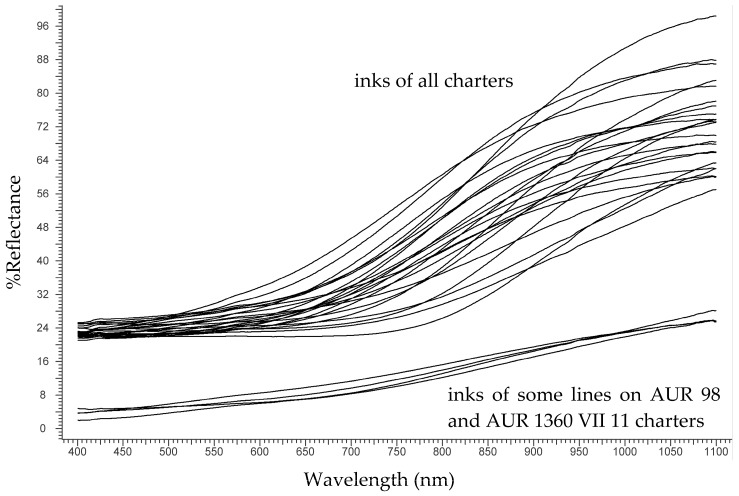
Fibre optic (FORS) spectra of the black inks.

FORS analysis of black inks allows obtaining a clear distinction between IGI on one hand and carbon-based, sepia and bistre inks on the other [4]. In the charters of the *Privilegium maius*, as expected, all the black inks analysed were of the IGI type (FORS spectra are shown in Figure 2) but in two charters, AUR 98 and AUR 1360 VII 11, part of the text was apparently written with a different ink (bottom spectra in Figure 2), most probably a mixture of IGI and carbon, as suggested by the lower reflectance in the Near Infrared NIR region.

It is possible that these lines of text were added later or by another scribe. In particular, one of such lines on AUR 98 charter contained the word TURRINBUOHC or Dürrenbuch, the place where the charter was issued. Table 1 shows the results of the analysis of the inks.

### 2.2. Analysis of the Coloured Threads—Non-Invasive Measurements

The coloured threads were analysed in order to identify the dyes and to have more significant information on the manufacture of the charters. The nature and the composition of the dyes could, in fact, reveal whether the threads were dyed (or not) at the same stage and with the same materials. Table 1 shows the thread colours analysed.

A first survey was carried out in situ with FORS and molecular spectrofluorimetry with fibre optic (FOMF) in the same analytical session as for the inks. For both analyses, the charters were simply laid on a table with the threads exposed to the probe of the instruments. Spectra obtained on yellow threads were not informative. Results for the other hues are discussed in the following sections.

#### 2.2.1. Purple Threads

All the charters contained purple threads; therefore, this hue is particularly informative. The combination of FORS and FOMF measurements allowed distinguishing the three groups:

1. One thread (AUR 1845) could be dyed with orchil, a lichen dye, as suggested by the two weak absorption bands between 540 and 590 nm [5];
2. The threads of AUR 187, AUR 520, AUR 708, AUR 1359 VIII 10, AUR 1360 VII 11 and possibly AUR 1844, could be dyed with anthraquinones from plants, according to the presence of two absorption bands between 500 and 540 nm [6];
3. One thread (AUR 1792) could be dyed with a modern colourant, considering the unusual spectral features and in particular the fluorescence emission at high wavelength. The term *modern* is to be referred to a re-dyeing process or to the substitution of the original thread with a modern one; indeed, we had no clue for the identification of the original purple colourant of this thread.

**Figure 3:**
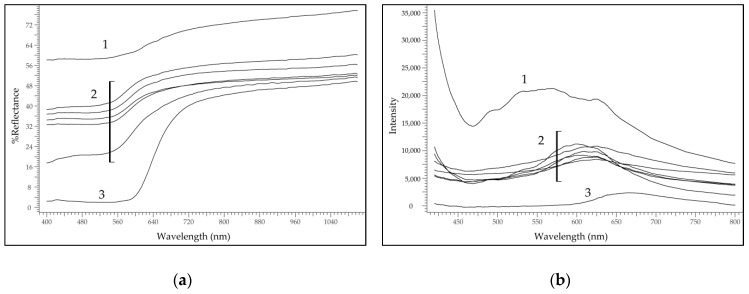
(**a**) FORS spectra from purple threads; (**b**) molecular spectrofluorimetry with fibre optic (FOMF) spectra from purple threads.

FORS and FOMF spectra from the purple threads are shown in Figure 3.

#### 2.2.2. Green Threads

The green threads of the charters AUR 1845, AUR 1359 VIII 10 and AUR 1360 VII 11, were apparently dyed using a double bath procedure by the superimposition of a yellow and a blue dye, as it was common in the Middle Ages because dyers could not rely on green dyes. In fact, FORS analysis showed in all instances the peak at ca. 650 nm that indicates the presence of indigo, the dye extracted from *Indigofera tinctoria* plant typical of South-eastern Asia, or woad, the dye extracted from *Isatis tinctoria* plant, native to Asia but widely diffused in Europe; it is not possible distinguishing them on the base of FORS analysis. Further investigation was needed to identify the yellow component.

#### 2.2.3. Preliminary Results

Table 2 summarises the results yielded by FORS/FOMF analysis. This preliminary information was used as a base for further investigation by more selective, micro-invasive techniques.

### 2.3. Analysis of the Coloured Threads—Micro-Invasive Approach

Few fibres (from a few mm to 1 cm in length) were taken from the threads in order to carry out measurements with Raman spectroscopy in conventional mode and in SERS mode, and with HPLC-DAD-MS. Sampling was necessary in order to address two main issues: (1) To use more selective techniques in order to obtain a complete characterisation of the dyes; (2) to investigate the possibility that threads had been re-coloured. This second aspect is suggested by the fact that FORS and FOMF are *superficial* techniques, so their spectral responses could come from the uppermost layer of dye applied to the thread; a sample, on the contrary, allows analysing all layers present on it, which is important in the case of subsequent dyeing or painting.

#### 2.3.1. Purple Threads

Firstly, conventional Raman analysis was used on the samples. This allowed the identification in the purple fibres of sample AUR 1792 the azo pigment Permanent Bordeaux FGR or PR14, patented in the first half of 20th century. The corresponding thread was apparently coloured in modern times.

Neither SERS spectra nor HPLC-DAD-MS were conclusive in identifying the colourants of the purple threads AUR 1844. Most probably, another modern colourant was used here.

The other samples of purple threads yielded useful spectra when analysed in SERS mode. The analysis was performed directly on-fibre or on extracts after treatment with concentrated formic acid. The difference among the two modes is that the former yields a response only from the molecules that are arranged on the surface with an orientation suitable to favour their interaction with the SER substrate. The extraction, on the other hand, allows the full characterisation of the dyes and is not prone to steric constraints. In both cases, 1 µL of the colloidal paste of silver nanoparticles was poured onto the sample—fibre or extract solution—and allowed to dry; after 10 min, the SER spectra were collected.

**Figure 4:**
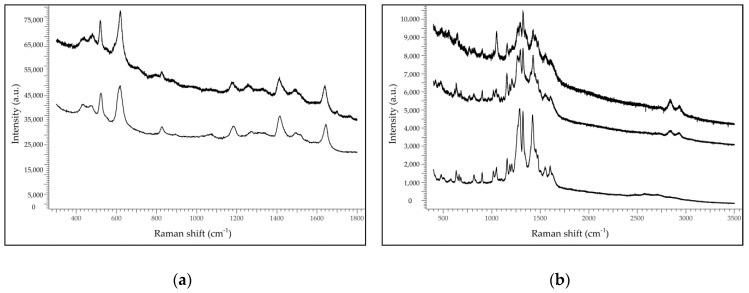
(**a**) Surface Enhanced Raman Spectroscopy (SER) spectra from purple threads of AUR 1845 (top) and standard orchil (bottom); (**b**) SER spectra from purple threads of AUR 187 (top), purple threads of AUR 520 (middle) and standard alizarin (bottom).

SERS analysis confirmed the grouping suggested by FORS/FOMF analysis, but yielded a more reliable identification, as it is based on the molecular fingerprinting of the dyeing molecules. The purple threads of AUR 1845 were apparently dyed with orchil, a lichen dye (SER spectra in Figure 4a). Orchil has been used since Greek-Roman times for painting and dyeing textiles [7]; many recipes, such as those contained in the Greek *Stockholm Papyrus* manuscript (3rd century AD) [8] cited it as a substitute for the more prized *Tyrian purple*, the famous dye obtained from mollusks, for example for colouring the parchment of purple codices (see [9] and references therein). In late Middle Ages, the Rucellai family from Tuscany resurrected the tradition of dyeing textiles with orchil, building their own wealth on the trade of lichen dyes.

Sample AUR 1845 was the only one dyed with orchil. In fact, the purple threads of AUR 187, AUR 520, AUR 708, AUR 1359 VIII 10 and AUR 1360 VII 11 were apparently dyed with *madder*, the natural dye extracted from the roots of *Rubiaceae* plants such as *Rubia tinctorum*, *Rubia peregrina* and *Rubia cordifolia*, or with (synthetic) alizarin, one of the main anthraquinones of madder (SER spectra in Figure 4b).

This is a first important result of SERS analysis: The purple threads of charters AUR 187, AUR 520 and AUR 708, belonging to the *Privilegium maius* group, were dyed with madder or synthetic alizarin, while the purple threads of the fourth charter AUR 1845 were dyed with a different colourant, i.e., orchil, a task apparently executed at a different stage.

The use of madder by dyers in Middle Ages was, by all means, common in the Mediterranean basin. However, it must be considered that alizarin has a high affinity for the silver nanoparticles [10], therefore it is not easy discriminating between the natural madder dye and modern synthetic alizarin, because SER spectra of the natural and of the synthetic dye show only slight differences [11]. The matter is of interest from a chronological perspective because, while madder was used since at least 2nd millennium BC, synthetic alizarin was firstly patented in 1868 by Graebe and Liebermann [12]. Considering the relevance of this aspect, the issue was further addressed by means of HPLC-DAD-MS.

The analysis was carried out on samples AUR 187, AUR 520 and AUR 708, determining the distribution of anthraquinones present in the purple dyes. The samples from the purple threads of AUR 1359 VIII 10 and AUR 1360 VII 11 were also included in this in-depth study. This analysis had two objectives: (1) Trying to determine the botanical source of madder—according to the literature, madder from *Rubia tinctorum* is particularly rich in alizarin, while madder from *Rubia cordifolia* and *Rubia peregrina* contains mostly purpurin, munjistin and pseudopurpurin [13,14]; and (2) verifying whether natural madder and/or synthetic alizarin were used.

**Figure 5:**
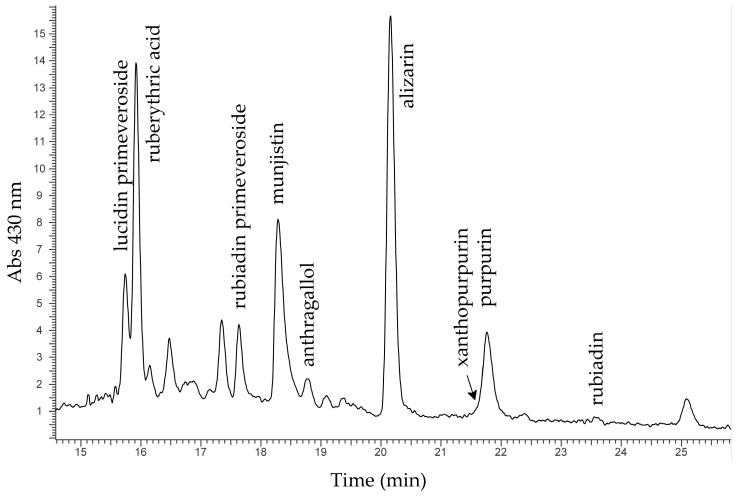
Chromatogram from a purple thread.

The results of HPLC-DAD-MS analysis suggested that the purple threads of AUR 187, AUR 520 and AUR 708 could be all dyed with madder from *Rubia tinctorum*, according to the distribution of the molecules identified with purpurin being much lower than alizarin. In addition, in all samples significant amounts of lucidin primeveroside and ruberythric acid (an alizarin precursor) were identified—these compounds are known to be contained in the roots of *Rubia tinctorum* [15,16,17]. A typical chromatogram from a purple thread is shown in Figure 5.

However, the chemical supplies of the madder dyes identified in samples AUR 187, AUR 520 and AUR 708 are not identical, as can be appreciated in Table 3 in which the relative amounts of alizarin, munjistin, purpurin and rubiadin, the main components of madder, are expressed after integration at 280 nm and normalisation of the peak areas to that of alizarin. The difference among the figures is in fact reasonably well above the expected variability for the analytical procedure.

This suggests that the purple threads of AUR 187, AUR 520 and AUR 708 were all dyed with madder from *Rubia tinctorum*, but not at the same stage or in the same dyeing bath, as already noted for the purple threads of AUR 1845, the fourth charter of the *Privilegium maius* group.

The purple threads of AUR 1359 VIII 10 and 1360 VII 11 were also dyed with madder, but in the case of AUR 1360 VII 11 the botanical source was most probably different from *Rubia tinctorum*, considering the high content of rubiadin [13] (Table 3).

The analysis of the purple threads of AUR 187, AUR 520 and AUR 708, as well as of AUR 1359 VIII 10 and 1360 VII 11, deserves a further consideration that is also the subject of the second objective of HPLC analysis. After a first chromatographic run carried out with an HPLC-DAD-MS system, analyses were repeated on a Ultra-High Performance Liquid Chromatography—tandem Mass Spectrometry (UHPLC-MS/MS) system, in order to verify the presence of chemical markers of synthetic alizarin, such as anthrapurpurin and flavopurpurin. In fact, according to the literature [18,19], these two compounds are known to be by-products of the synthesis of alizarin and, in addition, they do not occur in the species of the genus *Rubia* [20].

Anthrapurpurin and flavopurpurin are structural isomers of purpurin, i.e., they have the same quasi-molecular ion, and probably different MS/MS fragmentation of the purpurin itself. In order to dramatically increase the sensitivity towards the *m*/*z* signals of alizarin (240 u) and purpurin (256 u), and consequently also of anthrapurpurin (256 u) and flavopurpurin (256 u), a very sensitive and selective UHPLC-MS/MS method was developed. The increase of sensitivity was obtained using a UHPLC instrument equipped with a suitable UHPLC column packed with 1.8 μm particles. Moreover, the UHPLC hyphenation with the mass spectrometer that works in the most selective mode, such as selected reaction monitoring (SRM), guaranteed the best performances in terms of selectivity (only specific transitions Q1→Q3 are monitored in SRM mode). In order to further improve the sensitivity of the method, the MS worked in scheduled SRM, i.e., no information about the fixed dwell time of each transition is given, but only the retention time window in which each transition has to be monitored. The software automatically optimizes the maximum dwell time for each monitored transition improving reproducibility and sensitivity. Table 4 reports the selected *m*/*z* transitions monitored (four transitions for each anthraquinone compound) and the corresponding optimized electric parameters.

**Figure 6:**
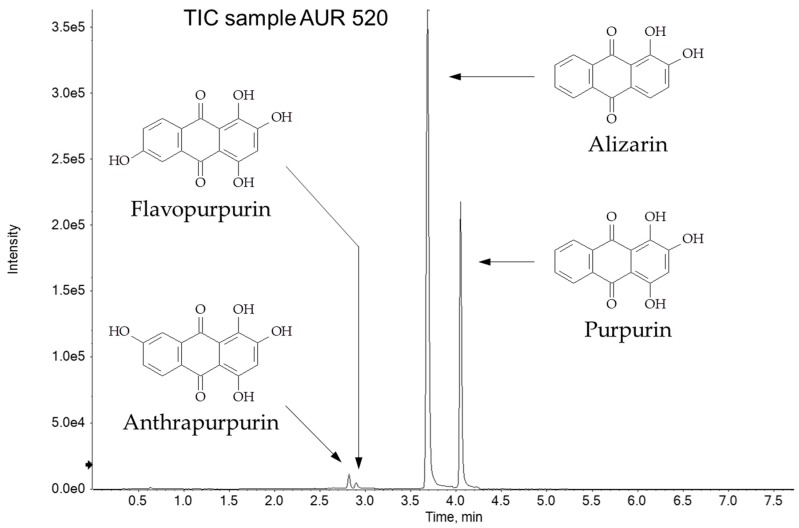
Total ion current chromatogram of the purple threads of AUR 520.

All the samples analysed, besides alizarin (t_R_ = 3.7) and purpurin (t_R_ = 4.1), contained two additional peaks eluting before the main anthraquinones. By comparison with a standard of synthetic alizarin, these peaks may be certainly attributed to anthrapurpurin (t_R_ = 2.8 min) and flavopurpurin (t_R_ = 2.9). The total ion current chromatogram of the purple threads of AUR 520 is shown in Figure 6, in which the fully resolved peaks of the four anthraquinones are shown, whereas the significant presence of the synthetic alizarin by-products is highlighted: anthrapurpurin and flavopurpurin signal intensities are 2.8% and 1.4% of alizarin, respectively.

These results indicate that the purple threads have been re-dyed in modern times. Moreover, the anthrapurpurin-to-flavopurpurin ratio, expressed in peak area, is between 2.0 and 2.4 for all threads, so it seems like re-dyeing occurred in a single stage.

#### 2.3.2. Yellow Threads

**Figure 7:**
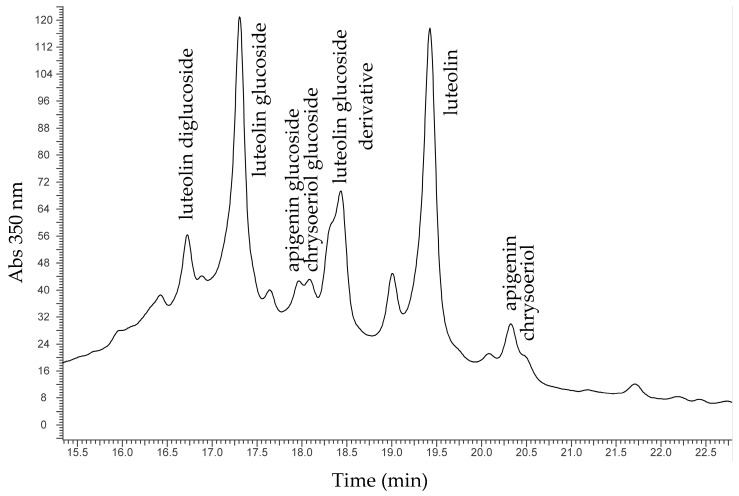
Chromatogram from a yellow thread.

It was not possible to obtain reliable SER spectra from the samples taken from yellow threads, so further investigation with HPLC-DAD-MS was carried out to identify the yellow dyes present on AUR 1792 and AUR 1845 threads. Sample AUR 1792 was not identified, so it can be hypothesised the presence of a modern synthetic colourant, in agreement with the use of PR14 for the purple threads. In the sample AUR 1845, after extraction with a mixture of formic acid/methanol/water (FMW) 2/1/1, HPLC-DAD-MS analysis yielded the identification of the chemical pattern typical of *weld* [21,22], the common natural dye obtained from *Reseda luteola* L., and used since Egyptian times. A representative chromatogram is shown in Figure 7, with most of the molecules identified being flavonoids.

Among the many different yellow dyes composed by flavonoids, the selective identification of weld is guaranteed by the presence of chrysoeriol [22].

#### 2.3.3. Green Threads

As with the yellow threads, the green threads of AUR 1845, AUR 1359 VIII 10 and AUR 1360 VII 11 charters did not yield conclusive SER spectra. Investigation with HPLC-DAD-MS provided information both on the yellow component and on the blue component, after extraction with specific solvents.

For the characterisation of the yellow component, samples were subjected to a first extraction in FMW mixture in order to extract flavonoids. In all cases, the presence of weld was identified, as for the yellow threads of AUR 1845. The very similar chemical supply would suggest that the green and the yellow threads of AUR 1845 were coloured starting from the same—or at least similar—dyeing bath.

As to the blue component of the green threads, preliminary investigation by means of FORS identified indigo or woad, but was not able to distinguish among them. This matter is relevant with concern to the geographic and historical point of view. In fact, while indigo was extracted from *Indigofera tinctoria*, a plant typical of South-eastern Asia, woad was extracted from *Isatis tinctoria*, a plant native to Asia but widely diffused in Europe. As suggested by a recent paper [23], the distinction between indigo and woad can be obtained according to the presence of isatin in relation to indigotin: a higher isatin/indigotin ratio would indicate *Indigofera tinctoria* as source. Samples of threads from AUR 1845, AUR 1359 VIII 10 and AUR 1360 VII 11 charters were then subjected to a second extraction in DMSO in order to release indigoid compounds. The results of the HPLC-DAD-MS analysis highlighted in all instances the identification of indigotin and indirubin, typical components of indigo and woad, but isatin was under the instrumental detection limit. This would point to the use of woad as the source of the blue colour.

## 3. Discussion

In the realm of the diagnostic campaign carried out on the charters of the *Privilegium maius*, the present study attempted to give a contribution to the knowledge of this controversial artefact by analysing the inks used for the text and the dyes used to impart colour to the threads.

### 3.1. Analysis of the Inks

The results of FORS analysis on the inks allowed the identification of iron gall inks (IGI) in all charters; in two cases, on charters AUR 98 and AUR 1360 VII 11, lines of text were a mixture of IGI and carbon. Apart this ostensible similarity, it is important to note that the simple identification of IGI does not in any way allow stating that the charters were written at the same stage because its use was by all means common in the Middle Ages. At the same time, it is not possible to date the charters on this base: in fact, IGI was used continuously from the late Antiquity to the 19th century, and there are not enough analytical data to provide a reference to a specific period, scriptorium or geographic area. Indeed, it can be only concluded that all the charters of the *Privilegium maius* were written with a similar ink. Further investigation is ongoing within the frame of a larger analytical campaign, using elemental techniques [24] in order to compare compositions and to highlight significant patterns [25].

### 3.2. Analysis of the Dyes Used for the Threads

By combining the results of non-invasive (FORS, FOMF) and micro-invasive (SERS, HPLC-DAD-MS) measurements, almost all the dyes were identified. The list is reported in Table 5.

It is obvious that the relative given dates refer to the dyeing processes only and cannot be further extrapolated to draw conclusions on the production of the threads or on the charters’ creation themselves. Despite this, some conclusions on the manufacture of the threads of the *Privilegium maius* charters can be suggested. Apart from the evidence of re-dyeing in AUR 187, AUR 520, AUR 708, AUR 1359 VIII 10 and 1360 VII 11 (synthetic alizarin), in AUR 1792 (Permanent Bordeaux FGR and unidentified yellow dye) and in AUR 1844 (unidentified purple dye), all the dyes identified are fully compatible with the presumed period in which the charters were produced, i.e., 14th century. Madder, weld and woad were among the most used dyes in medieval Europe. Orchil was again intensively used at the dawn of Renaissance after a tradition starting from Greek-Roman times.

The most relevant information deriving from the diagnostic study is that the purple threads of the four charters of the *Privilegium maius* (AUR 187, AUR 520, AUR 708 and AUR 1845) were apparently coloured at different dyeing stages rather than in a single stage. This could mean, perhaps, that they were prepared at different times.

A second relevant aspect is that all the purple threads of AUR 187, AUR 520, AUR 708, AUR 1359 VIII 10 and 1360 VII 11 have been apparently treated in modern times. Note that all these threads contained the typical supply of natural madder AND the typical markers of synthetic alizarin: it is then possible that these threads were originally dyed with natural madder from *Rubia tinctorum* and later on re-dyed with synthetic alizarin in modern times or at least after 1868. It is known that the wax seal of charter AUR 520 was accidentally broken and then inaccurately reassembled, perhaps during the preparation of the documents for a permanent exhibition at the Austrian State Archives which began in 1905. On that occasion, perhaps, it could have been decided to re-dye all the purple threads using synthetic alizarin, a dye already available for at least 35 years; it seems more reasonable to suggest *re-dyeing* rather than *replacing* the threads, as the HPLC-DAD-MS and UHPLC-MS/MS analyses evidenced the presence of the chemical markers of both natural madder and synthetic alizarin.

## 4. Materials and Methods

### 4.1. Reagents

Acetonitrile LC-MS Ultra Chromasolv (>99.9%) and standard of alizarin and purpurin were purchased from Sigma-Aldrich (Milwaukee, USA). A sample of Alizarin Red paste from The British Alizarine company was kindly provided by Dr. V.J. Chen (Indianapolis Museum of Art). Water UHPLC-MS grade was acquired from VWR Chemicals (Darmstadt, Germany). Nitric acid (69%), hydrochloric acid (37%), formic acid, methanol, DMSO, silver nitrate and sodium citrate dihydrate were purchased from Carlo Erba Reagents (Arese, Italy). Ultra-high quality (UHQ) water, employed throughout the experimental work for the preparation of solutions, was obtained by a Millipore (Darmstadt, Germany) Direct-q 3 system.

### 4.2. UV–Visible Diffuse Reflectance Spectrophotometry with Fibre Optic (FORS) Analysis

FORS analysis was performed with an Avantes (Apeldoorn, The Netherlands) AvaSpec-ULS2048XL-USB2 model spectrophotometer and an AvaLight-HAL-S-IND tungsten halogen light source; detector and light source were connected with fibre optic cables to an FCR-7UV200-2-1,5x100 probe. In this configuration, both the incident and detecting angles were 45° from the surface normal, in order not to include specular reflectance. The spectral range of the detector was 200–1160 nm; the overall operational range of the device (combination of lamp + detector) was 375–1100 nm. Depending on the features of the monochromator (slit width 50 µm, grating of UA type with 300 lines/mm) and of the detector (2048 pixels), the best spectra resolution was 2.4 nm calculated as FWHM. Diffuse reflectance spectra of the samples were referenced against the WS-2 reference tile provided by Avantes and guaranteed to be reflective at 98% or more in the spectral range investigated. The investigated area on the sample had a 1 mm diameter. The probe was inserted into an aluminium block, in order to exclude external light and to hold firmly the probe in place. During analysis, the block is laid on the sheet; therefore the side in contact with the manuscript was covered in Tyvek^®^, a soft tissue. In all measurements the distance between probe and sample was kept constant to 2 mm. To visualise the investigated area on the sample, the probe contained a USB endoscope inserted as well in the block. The instrumental parameters were as follows—10 ms integration time, 100 scans for a total acquisition time of 1.0 s for each spectrum. The whole system was managed by means of AvaSoft v. 8 dedicated software, running under Windows 7^TM^.

### 4.3. Molecular Spectrofluorimetry with Fibre Optic (FOMF) Analysis

Fluorescence spectra were obtained with an Ocean Optics (Dunedin, FL, USA) Jaz model spectrophotometer. The instrument is equipped with a 365 nm Jaz-LED internal light source; a QF600-8-VIS/NIR fibre fluorescence probe is used to drive excitation light onto the sample and to retrieve the emitted light. The spectrophotometer works in the range 191–886 nm; according to the features of the monochromator (200 µm slit width) and detector (2048 elements), the spectral resolution available is 7.6 nm calculated as FWHM. The investigated area on the sample is 1 mm in diameter. In all measurements, the sample-to-probe distance was kept constant at 6 mm, corresponding to the focal length of the probe. To visualise the investigated area on the sample, the probe contained a USB endoscope. Instrumental parameters were as follows: 2 s integration time, 3 scans for a total acquisition time of 6 s for every spectrum. The system was managed with SpectraSuite^TM^ software under Windows 7^TM^.

### 4.4. Raman Analysis

Raman analysis was performed with a high-resolution dispersive Horiba (Villeneuve d′Ascq, France) LabRAM HR model spectrophotometer coupled with a confocal microscope. The instrument is equipped with a 633 nm excitation laser, two (600 and 1800 lines/mm) dispersive gratings, an 800 mm path monochromator and a Peltier cooled CCD detector. The optical arrangement gave a spectral resolution of about 2 cm^−1^. Spectra were taken placing samples on the microscope stage and observing them with long working distance, 20×, 50× and 80× objectives. The sampled area was identified and focused using either a video camera or the microscope binoculars. Laser power at the sample was initially kept low (<100 μW) by means of a series of neutral density filters in order to prevent any thermal degradation of the molecules, then gradually increased to the optimal signal-to-noise ratio. Exposure time was 1–120 s according to individual needs. The system was managed with LabSpec 5 software running under Windows XP.

For SERS mode analysis, a colloidal paste of silver nanoparticles was prepared. Reagents and solvents (nitric acid, hydrochloric acid, methanol, silver nitrate and sodium citrate dihydrate) were purchased from Carlo Erba reagents (Arese, Italy); ultra high quality (UHQ) water was obtained by means of a Millipore (Darmstadt, Germany) Direct-q 3 system. The preparation of the silver colloidal pastes was carried out following a procedure described by Idone et al. [26] that modifies the method of Brosseau et al. [27]. SERS measurements were carried out both on-fibre and on the solution after extraction from the fibre with proper solvents. In the first case, 1 µL of colloidal paste was dropped on the sample and allowed to dry before exposing it to the laser beam; in the second case, 1 µL of colloidal paste was dropped on the solution extract, mixed with the tip of the pipette and the mixture was then allowed to dry before exposing it to the laser beam.

### 4.5. HPLC-DAD-MS Analysis

HPLC-DAD-MS analysis was carried out with an instrument equipped with two detection systems, a Diode Array Detector spectrophotometer (DAD) and a Mass Spectrometer (MS). The analytical conditions for HPLC-DAD-MS analysis were as follows: The instrument used was an Agilent 1100 HPLC-MSD Ion Trap XCT system, equipped with an electrospray ion source (HPLC-ESI–MS) (Agilent Technologies, Palo Alto, CA, USA). Separations were performed on a Jupiter C18 column 1 × 150 mm with 3-μm particle size (Phenomenex, Torrance, CA, USA). Eluents used were water (A) and acetonitrile (B), both added with 0.1% formic acid. The gradient employed was: 5% eluent B for 3 min, then linear to 95% eluent B in 25 min and finally hold at 95% eluent B for a further 15 min. The flow rate was set to 50 μL/min and the column temperature was set at 25 °C. The injection volume was 8 μL. Ions were detected in ion charged control with a target ion value of 200,000 and an accumulation time of 300 ms, using capillary voltage 3300 V; nebuliser pressure 15 psi; drying gas 8 L/min; dry temperature 325 °C; rolling averages 2; averages 5. Mass spectra were acquired in the negative ion mode in the 100–800 *m*/*z* range.

### 4.6. UHPLC-MS/MS Analysis

UHPLC/MS analyses were performed by Nexera Liquid Chromatography Shimadzu (Kyoto, Japan) system equipped by a DGU-20A3R Degasser, two LC-30AD Pumps, a SIL-30AC Autosampler, a CTO-20AC column compartment and a CMB-20A Lite system controller. The system was interfaced with a 3200 QTrap^TM^ LC–MS/MS system (Sciex, Concord, Canada) by a Turbo V^TM^ interface equipped with an ESI probe. The 3200 QTrap^TM^ data were processed by Analyst 1.5.2 software (Toronto, Canada). The stationary phase was an Acquity UPLC HSS T3 (2.1 × 100 mm, 1.8 µm) column (Waters, Milan, Italy). The mobile phase was a mixture of water with the addition of 1% of formic acid (A) and 90/10 (*v*/*v*) acetonitrile/methanol with the addition of 0.1% of formic acid (B), eluting at flow rate 0.600 µL min^−1^. The final gradient conditions of UHPLC-MS/MS working in selected reaction monitoring (SRM) mode were the following: 0.0–0.5 min 20% B, 0.5–1.9 min 50% B, 1.9–2.8 min 50% B, 2.8–4.0 min 99% B, 4.0–4.5 min 99% B, 4.6 min 20% until to 8.0 min. The injection volume was 10.0 µL and the oven temperature was set at 40 °C.

The turbo ion spray (TIS) ionisation was obtained using the Turbo V^TM^ interface working in positive ion (PI) mode. The instrumental parameters were set as follows: Curtain gas (N_2_) at 30 psig; nebuliser gas GS1 (N_2_) and GS2 (N_2_) at 70 and 75 psig, respectively; desolvation temperature (TEM) at 500 °C; collision activated dissociation gas (CAD) at 6 units of the arbitrary scale of the instrument and ion spray voltage (IS) at +5000 V. The mass spectrometer was used in scheduled multiple reaction monitoring (sMRM) mode considering the transitions of each analyte at a prefixed retention time (MRM detection window 30 sec and target scan time 1 s). Unit mass resolution was established and maintained in each mass-resolving quadrupole by keeping a full width at half maximum (FWHM) of about 0.7 µ.

### 4.7. Extraction Procedures

Different chemical solutions and conditions were used for the extraction, depending on the chemical natures of the dyes present on the threads. Extraction was obtained with the following procedures:

1. for purple threads, a mixture formic acid/methanol/water (FMW) 2:1:1 was used, 10 min at 90 °C;
2. for yellow threads and the yellow component of green threads, the same FMW mixture was used, 5 min at 80 °C;
3. for the blue component of green threads: DMSO, 10 min at 90 °C.

## 5. Conclusions

The analytical study on the dyes of the threads and on the inks used for the *Privilegium maius* was performed in the frame of the wide diagnostic campaign carried out at Österreichisches Staatsarchiv in Vienna. Though providing information on specific aspects, this study can help in assessing the very complex story of the charters.

As to the inks, analysis revealed that most of them were of the iron gall type, apart from those used in some parts of AUR 98 and AUR 1360 VII 11 charters that were probably iron gall/carbon mixtures.

As to the dyes, it was found that the purple threads were most probably dyed at different stages. The threads of three charters, i.e., AUR 187, AUR 520 and AUR 708 were dyed with natural madder obtained from *Rubia tinctorum*, but the threads of the charter AUR 1845 were apparently dyed with orchil, a lichen dye; in addition, HPLC-MS analysis revealed that the distributions of anthraquinones in the threads of AUR 187, AUR 520 and AUR 708 were significantly different, as if they were dyed with madder but in different dyeing baths or at different times. Finally, all the purple threads were apparently re-dyed in modern times, as they showed the chemical signature of synthetic alizarin, that is the contextual presence of flavopurpurin and anthrapurpurin.

In the end, even if this information cannot fix the time in which the charters of the *Privilegium maius* were produced, it suggests that they were possibly assembled at different times.

## Figures and Tables

**Table 1:** Lists the items analysed in this study.

Charter	Item Analysed	Group	Ink Type
AUR 98	ink	*Privilegium maius*	IGI ^1^, IGI ^1^/carbon
AUR 187	ink, dyed threads	*Privilegium maius*	IGI ^1^
AUR 520	ink, dyed threads	*Privilegium maius*	IGI ^1^
AUR 708	ink, dyed threads	*Privilegium maius*	IGI^1^
AUR 1845	ink, dyed threads	*Privilegium maius*	IGI ^1^
AUR 97	ink	comparative data	IGI ^1^
AUR 1359 VIII 10	ink, dyed threads	comparative data	IGI ^1^
AUR 1360 VII 11	ink, dyed threads	comparative data	IGI ^1^, IGI ^1^/carbon
AUR 1792	ink, dyed threads	comparative data	IGI ^1^
AUR 1844	dyed threads	comparative data	^2^

^1^ IGI: Iron Gall Ink. ^2^ ink not analysed.

**Table 2:** List of the coloured threads analysed.

Charter	Thread Colour	Identification by FORS/FOMF
AUR 187 ^PM^	purple	anthraquinone dye
AUR 520 ^PM^	purple	anthraquinone dye
AUR 708 ^PM^	purple	anthraquinone dye
AUR 1845 ^PM^	green	indigo/yellow dye
	purple	lichen dye
	yellow	n.i. ^1^
AUR 1359 VIII 10 ^cd^	green	indigo/yellow dye
	purple	anthraquinone dye
AUR 1360 VII 11 ^cd^	green	indigo/yellow dye
	purple	anthraquinone dye
AUR 1792 ^cd^	purple	modern dye
	yellow	modern dye
AUR 1844 ^cd^	purple	n.i. ^1^

^PM^*Privilegium maius*. ^cd^ comparative data. ^1^ not identified.

**Table 3:** Distribution of anthraquinone compounds in samples dyed with madder.

Compound	AUR 187	AUR 520	AUR 708	AUR 1845	AUR 1359 VIII 10	AUR 1360 VII 11
alizarin	100.0	100.0	100.0	^1^	100.0	100.0
munjistin	37.4	45.5	75.62	^1^	11.8	8.6
purpurin	4,8	1.9	21.5	^1^	16.4	9.9
rubiadin	45.5	12.1	1.1	^1^	2.8	119.7

^1^ not present.

**Table 4:** Scheduled Selected Reaction Monitoring (sSRM) positive ion transitions (Q1 and Q3 masses), time (min), and mass spectrometry parameters. The transitions are numbered from the most intense transition to the least one.

Compound	Q1	Q3	Time (min)	DP ^1^ (V)	EP ^2^ (V)	CE ^3^ V)	CXP ^4^ (V)
Alizarin 1	241.1	139.1	3.7	59.2	3.1	55.4	2.3
Alizarin 2	241.1	157.1	3.7	59.2	3.1	36.0	2.5
Alizarin 3	241.1	128.2	3.7	59.2	3.1	62.1	2.2
Alizarin 4	241.1	129.1	3.7	59.2	3.1	42.9	2.2
Flavopurpurin + Anthrapurpurin 1	257.1	173.1	2.8	62.0	10.0	36.7	2.8
Flavopurpurin + Anthrapurpurin 2	257.1	155.1	2.8	62.0	10.0	51.8	2.4
Flavopurpurin + Anthrapurpurin 3	257.1	127.1	2.8	62.0	10.0	60.4	2.2
Flavopurpurin + Anthrapurpurin 4	257.1	145.1	2.8	62.0	10.0	43.2	2.3
Purpurin 1	257.1	77.1	4.0	56.9	3.9	79.0	1.9
Purpurin 2	257.1	229.1	4.0	56.9	3.9	33.0	2.5
Purpurin 3	257.1	187.1	4.0	56.9	3.9	33.0	2.5
Purpurin 4	257.1	127.1	4.0	56.9	3.9	59.1	2.2

^1^ DP: Declustering Potential. ^2^ EP: Entrance Potential. ^3^ CE: Collision Energy. ^4^ CXP: Collision cell eXit Potential.

**Table 5:** Final identification of the dyes.

Charter	Thread Colour	Final Identification	Possible Dyeing Period
AUR 187	purple	(1) madder from *Rubia tinctorum* (2) synthetic alizarin	(1) medieval (2) modern (19th century)
AUR 520	purple	(1) madder from *Rubia tinctorum* (2) synthetic alizarin	(1) medieval (2) modern (19th century)
AUR 708	purple	(1) madder from *Rubia tinctorum* (2) synthetic alizarin	(1) medieval (2) modern (19th century)
AUR 1845	green	woad/ weld from *Reseda luteola*	medieval
	purple	orchil	medieval
	yellow	weld from *Reseda luteola*	medieval
AUR 1359 VIII 10	green	woad/weld from *Reseda luteola*	medieval
	purple	(1) madder from *Rubia tinctorum* (2) synthetic alizarin	(1) medieval (2) modern (19th century)
AUR 1360 VII 11	green	woad/weld from *Reseda luteola*	medieval
	purple	(1) madder from *Rubia tinctorum* (2) synthetic alizarin	(1) medieval (2) modern (19th century)
AUR 1792	purple	Permanent Bordeaux FGR	modern (20th century)
	yellow	n.i. ^1^	possibly modern
AUR 1844	purple	n.i. ^1^	possibly modern

^1^ not identified.
